# Pesquisa antimanicomial com familiares de vítimas da violência policial

**DOI:** 10.1590/0102-311XPT031224

**Published:** 2024-06-17

**Authors:** Martinho Braga Batista e Silva

**Affiliations:** 1 Instituto de Medicina Social, Universidade do Estado do Rio de Janeiro, Rio de Janeiro, Brasil.

No livro *Na Mira do Fuzil: a Saúde Mental das Mulheres Negras em Questão* ^1^, a autora entrevistou cinco mulheres negras e teceu uma série de considerações sobre a política de saúde mental no Brasil a partir desse diálogo, destacando cinco processos que atravessaram essas e outras vidas vitimadas pela violência policial no Rio de Janeiro: patologização, psicologização, psiquiatrização, medicalização e farmacologização. O livro é composto de cinco capítulos, todos eles com títulos aspeados; quando chegamos ao terceiro - *Mães de Bandidos* -, obtemos acesso às falas das entrevistadas, tendo seus nomes substituídos por flores para manter a confidencialidade: Cravo, Girassol, Margarida, Orquídea e Rosa. Com o objetivo de “*analisar os impactos da violência armada na saúde mental das mulheres negras, dando destaque ao processo de sofrimento e adoecimento psíquico ocasionados pela mutilação ou morte de seus filhos*” (p. 19), a pesquisa qualitativa foi desenvolvida “*ao longo de cinco anos, sendo fruto de um conjunto de ações de extensão, orientações, reflexões, troca e demais atividades realizadas*” (p. 21-2). As fases que essas mães moradoras de favelas vivenciaram para elaborar sua dor também são cinco: desespero; choque; perda de rumo; questionamentos; e deslocamento ou permanência. Além disso, o campo da atenção psicossocial desenhado pela autora é composto de cinco dimensões: acadêmico-científica; ético-política; jurídico-legislativa; técnico-operacional; e sociocultural. Finalmente, a publicação do livro antecedeu a 5ª Conferência Nacional de Saúde Mental, em dezembro de 2023, momento no qual a autora já fazia parte da equipe do Departamento de Saúde Mental, Álcool e outras Drogas do Ministério da Saúde. Desse modo, vou me permitir estruturar a resenha do livro em cinco tópicos, procurando destacar suas contribuições para o campo da saúde mental.

Quem são as entrevistadas? Comentando um relatório oficial publicado em 2021, a autora do livro afirma que ele “*comprova que a população negra é mais do que o dobro de brancos em situação de extrema pobreza, sendo as mulheres negras as que mais apresentaram incidência de pobreza e extrema pobreza*” (p. 41). Em diálogo com Davis, Kilomba, Lugones, Flauzina, Hooks e diversas outras autoras, Passos demonstra que propaga-se no imaginário social a “*concepção de que as mulheres negras possuem uma capacidade de suportar a dor muito maior do que as outras*” (p. 43), de maneira que haveria uma naturalização de uma suposta fragilidade feminina, ao mesmo tempo em que ela nunca foi reconhecida para as mulheres negras, reduzidas ao lugar de fêmea, coisificadas e animalizadas. Além disso, sublinha a revisão de literatura apontando que “*a exposição ao estresse ocasionado pelas discriminações pode ser um mecanismo causal para transtornos mentais*” (p. 47). Com base nessa ampla fundamentação teórica, os 21 trechos das falas das interlocutoras espalhados no terceiro e quarto capítulos ganham significado: Rosa começa a entender que pode “*viver o luto sem ser no cantinho*” (p. 105), enquanto Orquídea revela que, nos dias em que as operações policiais na favela acontecem com helicópteros, “*já chega com tiros* (...) *não consigo fazer minhas tarefas mais corriqueiras, as tarefas mais práticas, não consigo comer*…” (p. 104). Margarida explica o manejo da dor nesse cenário, cercado de automutilações, ao mesmo tempo em que Girassol destaca a insônia e Cravo, a peculiaridade da morte destinada às mães de filhos vítimas de violência policial letal: “*mata a gente aos poucos*” (p. 76). As cinco interlocutoras mencionam outras duas, Violeta e Petúnia, para exemplificar situações nas quais sintomas físicos e mentais emergem após a morte dos filhos por violência policial, adoecendo e incapacitando mães negras moradoras de favela.

Qual é o método? Há bastante tempo, um padrão em pesquisas qualitativas foi denunciado: entrevistas semiestruturadas, como procedimento de coleta, e análise de conteúdo temática, como procedimento de análise dos dados, além de percepções e/ou sentidos sobre um processo saúde-doença como objeto de estudo genérico, entre outros elementos que apontavam para uma falta de correspondência entre teoria e metodologia adotadas, fragilizando a subárea de Ciências Sociais e humanas em saúde no interior da área de Saúde Coletiva ^2^. Passos não adota uma metodologia convencional, já que combina entrevistas individuais e observação participante de eventos com a finalidade de “*descortinar as bases racistas e colonialistas que fundamentam a psiquiatria e incidem no cuidado em saúde mental*” (p. 19). Além disso, as análises sobre o material coletado se encontram amparadas em uma vasta bibliografia sobre as relações entre racismo, psiquiatria e direito, inclusive sobre guerra, como se pode notar no diálogo com Martin-Baró e Mbembe.

Qual é o contexto no qual as interlocutoras foram entrevistadas? Um dos pontos que merecem maior aprofundamento diz respeito à pandemia de COVID-19, já que a investigação ocorreu justamente durante esse período - o projeto de pesquisa aprovado pela Câmara de Ética em Pesquisa é do ano de 2022 - e os poucos momentos nos quais o impacto dessa crise sanitária é mencionado (p. 21, 47 e 114) não permitem notar em que medida ela atingiu as entrevistadas e a própria condução do estudo.

Quais são os processos sociais em curso nesse contexto? A farmacologização apontada pela autora remete ao fato de que “*aciona-se como resposta o uso de fármacos - sendo eles prescritos ou não pelos médicos -, porém utilizados largamente pela população para alívio das dores promovidas pela guerra*” (p. 72), sendo que identificar se houve ou não a atividade de prescrição, bem como se o que foi prescrito é um fármaco ou um psicofármaco, é fundamental para distinguir esse processo da medicalização e da psiquiatrização, de maneira que esses e outros conceitos - eurocêntricos, por sinal - demandam maior precisão em seu uso para caracterizar o fenômeno investigado. Rosa, interlocutora cujos trechos de fala aparecem com maior frequência no livro, é contundente sobre a farmacologização: “*Esse tomar remédio já se inicia no cemitério. Muito mesmo das colegas que dão…*” (p. 87).

Quais lutas antimanicomiais, atenções psicossociais e reformas psiquiátricas estão no horizonte da autora-pesquisadora? A autora denuncia uma “*certa concepção hegemônica de saúde mental que é sustentada pela psiquiatria clássica* (...) *que afirma a manutenção das hierarquias raciais, sociais e generificadas através da patologização, medicalização e farmacologização das existências…*” (p. 92) e sustenta que “*a atenção psicossocial só pode existir sendo antimanicomial, antirracista e decolonial*” (p. 117). Além disso, não poupa críticas a uma certa “*noção de que a Luta Antimanicomial é restrita à micropolítica*” (p. 97), partindo na direção das chamadas práticas palmarinas.

Embora todos os títulos dos capítulos sejam aspeados, nenhum deles expressa um trecho de fala de uma entrevistada, recortando fragmentos de pronunciamentos de governadores publicados em jornal, títulos de livros de notórios pioneiros da luta antimanicomial, entre outros documentos. Inclusive, logo no primeiro capítulo, notícias de jornal apresentaram Janaína, Adriana e Joselita, também mães cujos filhos foram vítimas de violência policial, depois mais trechos de jornal abordam esse tipo de violência por meio dos casos George Floyd e João Pedro. Quais as diferenças entre essas histórias contadas pela imprensa e aquelas contadas pelas participantes da pesquisa? Um balanço entre o material documental e o material relativo à interação que ampara a pesquisa pode suscitar futuras publicações dessa brilhante autora-pesquisadora-gestora da política antimanicomial.
